# Understanding the motivational mechanisms behind the usage frequency of ride-hailing during COVID-19 pandemic

**DOI:** 10.3389/fpubh.2022.1097885

**Published:** 2023-01-26

**Authors:** Shuai Ling, Yunqi Jia, Xuemin Yuan, Hongming Dong, Tianjing Zhang

**Affiliations:** ^1^College of Management and Economics, Tianjin University, Tianjin, China; ^2^Laboratory of Computation and Analytics of Complex Management Systems (CACMS), Tianjin University, Tianjin, China; ^3^TOEC Technology Co., Ltd., Tianjin, China

**Keywords:** ride-hailing service, passengers' trust and loyalty, usage frequency, exploratory factor analysis, COVID-19 pandemic

## Abstract

**Introduction:**

This study aimed to explore the factors influencing people's utilization of ride-hailing services, particularly in the context of the COVID-19 pandemic.

**Methods:**

A two-stage survey was conducted among the same group of passengers pre and post COVID-19 pandemic, resulting in a total of 670 valid samples. Exploratory factor analysis (EFA) was applied to the data, followed by the ordered probit and ordered logit models to identify the motivational factors behind passengers' frequency of using ride-hailing.

**Results:**

The findings indicated that trust and loyalty were the most influential factors in determining passengers' frequency of using ride-hailing services. However, passengers' perception of the COVID-19 pandemic did not have a significant effect on the frequency of using ride-hailing.

**Discussion:**

This research provides empirical evidence and policy implications for understanding people's usage of the ride-hailing services in the context of public-health emergency.

## 1. Introduction

The utilization of mobile technology has had a considerable impact on the form of transportation services and the way passengers travel (1, 2). For instance, passengers can now use ride-hailing services to request a driver to pick them up at a specified location (3–6). This type of service is beneficial as it can save time for both passengers and drivers, as well as increase vehicle efficiency. Consequently, ride-hailing services have been adopted by many companies globally, such as Uber and Lyft (USA), Didi Chuxing (China), Ola (India), and Grab (Southeast Asia). Statistics indicate that ~3.2 billion passengers have used ride-hailing services, which is comparable to the number of people using urban bus and rail systems (7). However, there are also divergent findings. For example, Pew Research Center (8) revealed that only 3% of their sample data (*N* = 4,787) used ride-hailing services on a daily basis, while the remaining 12% used these services once a week.

Studies found numerous factors influence passengers' ride-hailing frequency, for example: psychological factors. Septiani et al. (9) found factors of internal perception and innovation characteristic influence the behavioral intention of online transportation service. Similarly, Huynh et al. (10) found attitude and subjective norms influence passengers' intention to use Uber/Grab services.

### 1.1. Social factors

Nguyen-Phuoc et al. (11) found trust fully mediate the relationships between perceived booking app-related risks and satisfaction and loyalty.

### 1.2. Demographics factors

The female passengers are 28.51% higher than that of male passengers who use ride-hailing services (10). Older ride-hailing passengers make more transit trips than others (12). Alemi et al. (13) found that passengers with higher educational and income levels were more likely to utilize these services, due to their familiarity with new technologies and other attributes of vehicle ownership. Murphy and Feigon (14) showed that the most frequent users of on-demand ride-hailing services were those from middle-income families with annual incomes between 50,000 USD and 75,000 USD. Those who have a strong inclination toward using their own vehicle tend to use ride-hailing services less frequently (15).

### 1.3. Environment factors

There is a close relationship between different building environments and passengers' ride-hailing frequency. For example, building density has a significant inhibitory effect on ride-hailing trips (16). Moreover, environmental consciousness plays an important role in ride-hailing frequency of use (17).

However, so far, there have been limited studies on the factors affecting the usage frequency of ride-hailing services in developing countries, especially the quantitative analysis of the potential impact of these factors. In addition, the continuous success of ride-hailing services is accompanied by various challenges, such as passengers' trust and loyalty, which are essential for these companies in terms of market share and profits. The concept of customer loyalty was first proposed by Guest (18), referring to customers' long-term psychological attachment to the company's products. Later, Boulding et al. (19) showed that loyalty is expressed in customers' higher willingness to recommend the company's supplies. Ride-hailing services have the potential to reduce traffic and emissions by reducing car ownership (20, 21). However, the impact of passengers' trust and loyalty on the frequency of ride-hailing usage has not been fully explored (22). Studies have shown that trust can increase people's intention to use such services (23, 24). Previous research has mainly focused on passengers' trust and loyalty prior to adoption (25). There are discrepancies between passengers' initial adoption and their actual usage frequency (26–28). For example, passengers' initial adoption can be altered over time (28). Thus, it is essential for ride-hailing companies to understand the influence of trust and loyalty on the frequency of using ride-hailing services in order to meet passengers' needs.

Additionally, due to the COVID-19 pandemic, another new challenge arises for ride-hailing: the social environment changed dramatically. After the outbreak of the COVID-19 pandemic, the World Health Organization (WHO) encourages people to maintain physical distance to avoid the epidemic spreading. In December 2019, after the outbreak of COVID-19, the Chinese government encouraged residents to minimize travel, for security reasons. The transportation services market has shrunk significantly, and ride-hailing is inevitably affected (29). In addition, safety concerns during the pandemic become the key consideration of transportation services (30). Passengers' behavioral patterns including the usage frequency of ride-hailing changed under the influence of COVID-19 pandemic (31). Therefore, to better understand the passengers' behavioral change, this study conducted a two-stage survey among the same group of passengers in pre and post COVID-19 pandemic.

Overall, this study attempts to addresses the following questions:

(1) Do the passengers' trust and loyalty significantly affect the usage frequency of ride-hailing?
(2) What is the effect of COVID-19 pandemic on passengers' usage of ride-hailing?
(3) What other factors will affect how often people use ride-hailing?

**Figure 1:**
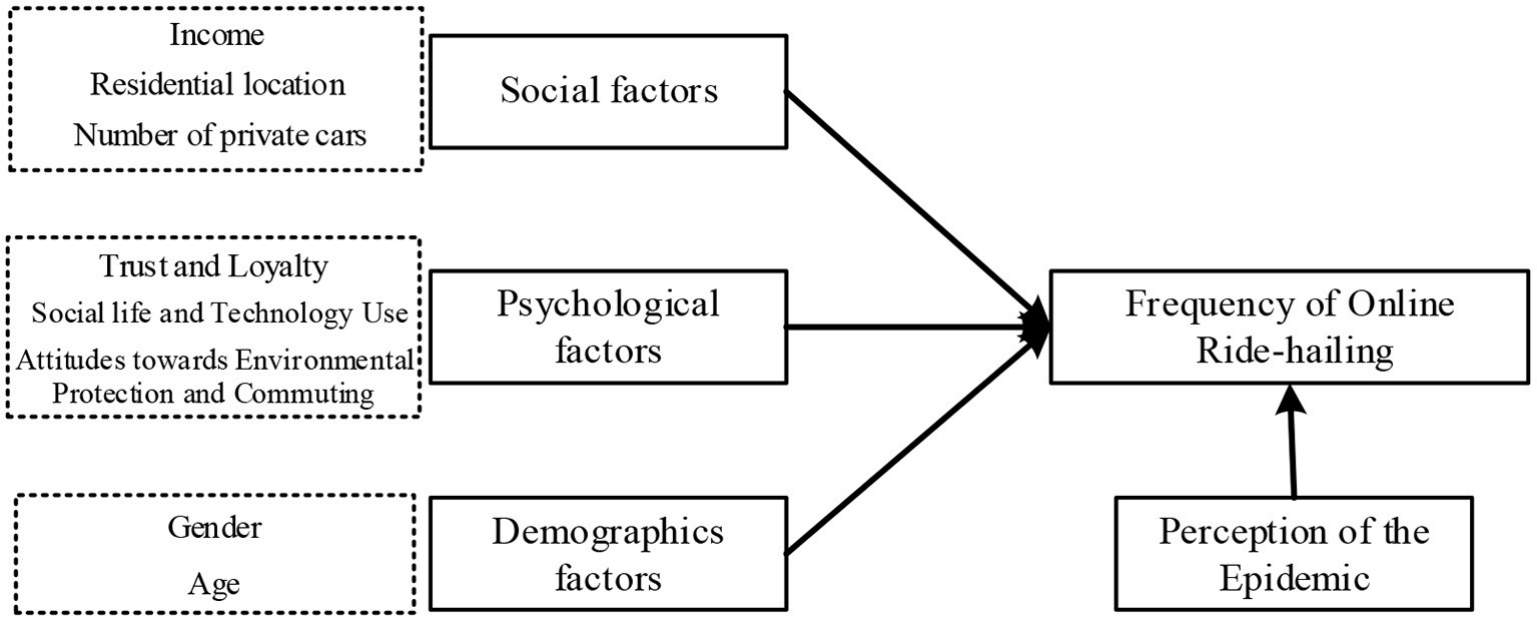
Typical factors selected in this research.

In order to answer these questions, this research proposes a theoretical model (as shown in Figure 1) based on Kotler (32) in order to construct and validate a conceptual framework to explain the usage frequency of ride-hailing. Specifically, the exploratory factor analysis (EFA) is conducted to develop the ordered probit and ordered logit models to study the affecting factors of the usage frequency of ride-hailing. Methods section introduces the data and methods. Results section introduces the models' construction details and analysis results. Discussion section discusses the findings. Finally, Conclusion section draws the conclusions.

## 2. Methods

### 2.1. Data collection and descriptive statistics

**Figure 2:**
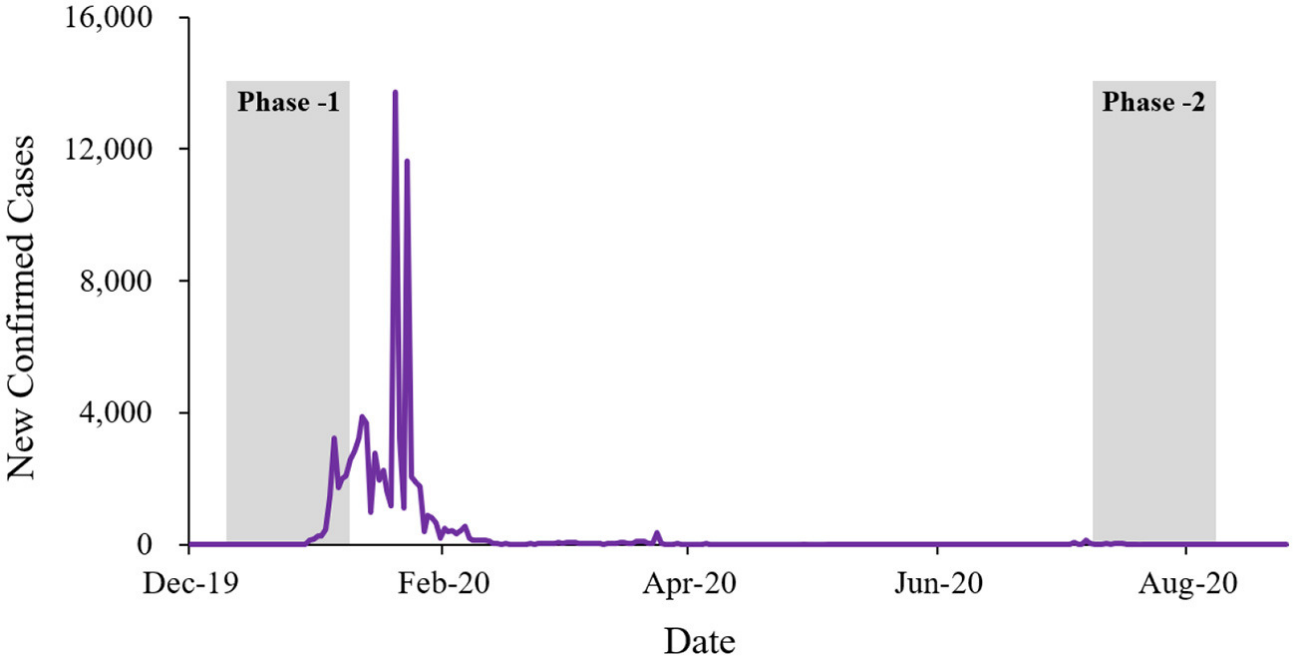
The new confirmed cases of COVID-19.

The questionnaire survey was conducted through Sojump, a popular online survey platform in China, following a pre-test with 30 respondents to ensure its applicability. The survey was divided into two phases: the pre-epidemic phase (from December 2019 to January 2020, Phase-1), where COVID-19 was not yet widespread in China, and the post-epidemic phase (from August 2020 to September 2020, Phase-2), where the number of new cases per day was less than 20 and mostly imported from abroad, indicating the effective control of the epidemic. Figure 2 shows the newly confirmed cases of COVID-19 during the survey period in China.

This two-stage survey was conducted among the same group of participants. Initially, 800 questionnaires were collected in the Phase-1 survey. When the epidemic was under control, the Phase-2 survey was conducted, resulting in 694 questionnaires. After eliminating 24 ineffective questionnaires, 670 valid samples were recruited. The participants were asked to answer questions related to the usage of ride-hailing, including whether they had used ride-hailing, followed by social factors, psychological factors, personal factors and perception of the epidemic. The psychological factors and perception of the epidemic were measured using a five-level Likert scale, with “1” indicating “strongly disagree,” “3” indicating “neutral,” and “5” indicating “strongly agree”.

The descriptive statistics about the sample are presented in Table 1. Participants included both students and the working population, which accounted for 39.48 and 57.49%, respectively. Males constitute 61.38% and females represent 38.62%. According to the report released by Aurora Big Data, in China, most of the users of independent ride-hailing apps were male, and the proportion of male users of Shenzhou Special Car and Shouqi's ride-hailing apps has been close to 70%. Didi Chuxing has a relatively high proportion of female users of 41.1%. In addition, according to the report released by Aurora Big Data, Beijing, Hangzhou, Wuhan, Guangzhou, Dalian, Tianjin, Chengdu, Shenyang, Shenzhen, and Hefei have been the top ten most popular cities for ride-hailing in China. In our survey, participants from these ten cities accounting for 67% which indicate that the participants in our survey have a certain degree of representativeness.

**Table 1:** Demographics and socioeconomics of the sample.

**Categorical variable**	**Category**	**Frequency**	**Percent (%)**
Gender	Male	414	61.79
	Female	256	38.21
Age	Under 18	12	1.79
	18–24	338	50.45
	25–34	278	41.49
	35–41	33	4.93
	42 and above	9	1.34
State of life	Student	264	39.40
	Worker	391	58.36
	Else	15	2.24
Income per month (Yuan)	< 3,000	208	31.04
	3,000–6,000	176	26.27
	6,000–10,000	208	31.04
	10,000–20,000	61	9.10
	>20,000	17	2.54
Education	Junior High school and below	18	2.69
	High School	147	21.94
	Bachelor's degree and junior college	335	50.00
	Post-graduate and above	170	25.37
Number of private cars	0	165	24.63
	1	435	64.93
	2 and above	70	10.45
Have Children (under 14 years old)	Yes	342	51.04
	No	328	48.96

### 2.2. Model development

#### 2.2.1. Exploratory factor analysis

The use of Exploratory Factor Analysis (EFA) was employed to reduce the dimensions of the collected data. Studies have demonstrated that when multiple measurement variables are used to represent a common factor, EFA can provide more accurate results (33, 34). EFA is a method used to gain a better understanding of the relationships between a set of measured variables by determining the number and type of common factors that explain the correlation patterns. Thus, EFA can integrate variables with complex relationships into a few core factors.

#### 2.2.2. Ordered probit model and ordered logit model

In regression models, the dependent variable is usually measured by a ratio scale. However, when the dependent variable is binary, sequence, or identifier, the ordinary least square method is no longer the best-unbiased estimator. Therefore, the ordered logit and ordered probit models in the discrete choice model are used to estimate the regression model of multiple ordered variables. These models are based on maximum likelihood estimation and assume that the random disturbance follows either a logistic or a multivariate normal distribution. Although the ordered probit model is more attractive in theory, it is difficult to prove that the dependent variable follows a normal logit distribution function or a standardized normal distribution function strictly. Thus, two regression methods are applied to the collected data to obtain a more reliable conclusion by comparing their results.

The basic principle of the ordered logit and ordered probit models is that there is an unobserved continuous variable Y^*^that satisfies the following relation:


(1) $$\begin{array}{l} Y^{*}=\sum \beta_{j}X_{j}+\varepsilon , \end{array}$$

where *Y* denotes the latent variable indicating the frequency of people using the ride-hailing; *X*_*j*_ denotes the factor score of the impact factor obtained by the EFA; β_*j*_ denotes the unknown parameter that needs to be estimated; and ε represents the random error term. In this work, it was assumed that the mean of all error terms was zero and the error terms of different subjects were irrelevant. The relationship between the observed ordered variable *Y* and Y^*^ is as follows:


(2) $$Y=\{\begin{array}{c} 1\;if\;-\infty <Y^{*}<k_{1}\\ 2\;if\;k_{1}<Y^{*}<k_{2}\\ 3\;if\;k_{2}<Y^{*}<k_{3}\\ 4\;if\;k_{3}<Y^{*}<k_{4}\\ 5\;if\;k_{4}<Y^{*}<\infty \end{array}\;.$$

## 3. Results

### 3.1. Reliability test and EFA results

In order to reduce the influence of individual choice differences on the results, subjects whose current city and urban residential location remained constant between the two phases of the study were chosen. It was also determined that the time gap between the two questionnaires was too short for personal preferences to change significantly. Additionally, Cronbach's alpha coefficient of the questionnaires was calculated to be 0.953 and 0.954 for the first and second phases respectively, indicating a high reliability. Furthermore, the KMO values of the first and second phases were 0.949 and 0.950 respectively, both of which were larger than 0.7, thus demonstrating the suitability of the questionnaires for further analysis using EFA.

Subsequently, EFA was employed to identify independent regression variables and calculate factor scores. After data cleaning and preprocessing, the number of factors influencing personal attitudes before the epidemic was reduced from 49 to 23, which were organized into three groups: Attitudes to Environmental Protection and Commuting (ATT), Social Life and Technology Use (SOC), and Passenger Trust and Loyalty (TRU). The latter refers to the passengers' loyalty to the ride-hailing service, not the third-party platforms providing the service. Similarly, the number of factors influencing personal attitudes after the epidemic was reduced from 53 to 29, which were organized into four groups: Attitudes to Environmental Protection and Commuting (ATT), Social Life and Technology Use (SOC), Passenger Trust and Loyalty (TRU), and Epidemic Impact (EPI). The results of the rotated component matrix before and after the epidemic are presented in Supplementary Tables A2, A3 in the Appendix, respectively, with factor loading coefficient values all greater than 0.5.

### 3.2. Results of ordered probit and ordered logit models

The ordered probit regression and an ordered logit regression were conducted on all the factors in the above analysis that affected the usage frequency of ride-hailing services in the pre-epidemic and post-epidemic stages. The regression results showed that in the pre-epidemic and post-epidemic stages, seven factors had significant effects on the frequency of use of the dependent variable. It is worth noting that in the post-epidemic stage, the EPI factor did not have a significant effect on the dependent variable. The comparison results of regression models before and after the epidemic are presented in Table 4.

The regression model can be explained based on the results of the ordered logit and ordered probit regression, which are shown in Table 2. The results showed that people's usage frequency of ride-hailing services was significantly affected by the following seven independent variables: ATT, SOC, TRU, AGE, INC, CAR, and LOC. The comparison of the regression coefficients before and after the epidemic shows that the influences of various factors on the dependent variable before and after the epidemic did not change significantly in the coefficients. However, after the epidemic, the influence of passengers' trust and loyalty on the usage frequency of ride-hailing significantly increased, while the influences of social life and technology significantly decreased.

**Table 2:** Comparison of the regression model coefficients before and after the epidemic.

**Factor**	**Ordered logit**		**Ordered probit**	
	**Before**	**After**	**Before**	**After**
ATT	0.8884^***^ [0.0899]	0.9427^***^ [0.0938]	0.5041^***^ [0.0502]	0.5337^***^ [0.0518]
SOC	0.2205^***^ [0.0801]	0.1722^**^ [0.0798]	0.1174^**^ [0.0463]	0.0925^**^ [0.0459]
TRU	0.5366^***^ [0.0876]	0.5991^***^ [0.0886]	0.3058^***^ [0.0508]	0.3464^***^ [0.0504]
EPI		0.0616 [0.0892]		0.0266 [0.0510]
AGE	−0.2379^**^ [0.1180]	−0.2344^**^ [0.1185]	−0.1161^*^ [0.0685]	−0.1206^*^ [0.0689]
GEN	−0.2290 [0.1600]	−0.1906 [0.1602]	−0.1425 [0.0930]	−0.1239 [0.0932]
INC	0.7955^***^ [0.0891]	0.7672^***^ [0.0887]	0.4332^***^ [0.0496]	0.4187^***^ [0.0493]
CAR	0.2482^**^ [0.1244]	0.2440^**^ [0.1247]	0.1427^*^ [0.0731]	0.1386^*^ [0.0733]
LOC	−0.5005^***^ [0.1631]	−0.5172^***^ [0.1643]	−0.2818^***^ [0.0945]	−0.2975^***^ [0.0948]
k1	−2.4205 [0.5301]	−2.5024 [0.5308]	−1.3433 [0.3059]	−1.4213 [0.3071]
k2	−0.5853 [0.5223]	−0.6179 [0.5228]	−0.3187 [0.3037]	−0.3713 [0.3044]
k3	0.9922 [0.5216]	0.9752 [0.5219]	0.5915 [0.3036]	0.5484 [0.3041]
k4	2.5018 [0.5275]	2.4795 [0.5276]	1.4691 [0.3056]	1.4258 [0.3059]

Std.Err is in the brackets. ^*^p < 0.1; ^**^p < 0.05; ^***^p < 0.01.

The main reason why individuals' perception of the epidemic was not a significant affecting factor was that, in the second stage of the questionnaire, the epidemic situation in China had been effectively controlled, work and production had been resumed in an orderly manner in various regions, and travel restrictions had gradually weakened. In the long run, the authors believe that the usage frequency of ride-hailing services may no longer be affected by the epidemic in the future. However, the emergence of the epidemic has brought higher requirements for the safety of ride-hailing services, such as requiring daily disinfection and requiring passengers and drivers to wear masks. Therefore, in the second phase of the questionnaire, the influence of passengers' trust and loyalty on the usage frequency of ride-hailing increased significantly. Due to restrictions on travel during the epidemic, people's social and entertainment activities were reduced during the investigation phase, so the impact of social life and technology use on the usage frequency of ride-hailing in the post-epidemic phase was significantly weakened.

### 3.3. Model verification

The goodness of fit of the model was tested. The test results of the two models before and after the epidemic are presented in Table 3. The result of the chi-square test shows that *P*(Sig.) was less than 0.001, which was statistically significant, illustrating that our model was meaningful as a whole. The values of R^2^ and Log likelihood of the two models were similar, indicating that there was no obvious difference between the advantages and disadvantages of the two models.

**Table 3:** Model fit of research models.

**Stage**	**Model**	**LR chi^2^**	**Pseudo R^2^**	**Log likelihood**	**Sample size**
Before	Ordered logit	380.09^***^	0.1787	−873.1602	670
	Ordered probit	392.15^***^	0.1844	−867.1308	670
After	Ordered logit	370.34^***^	0.1742	−878.0359	670
	Ordered probit	383.67^***^	0.1804	−871.3674	670

^***^p < 0.01.

### 3.4. Marginal effect analysis

**Figure 3:**
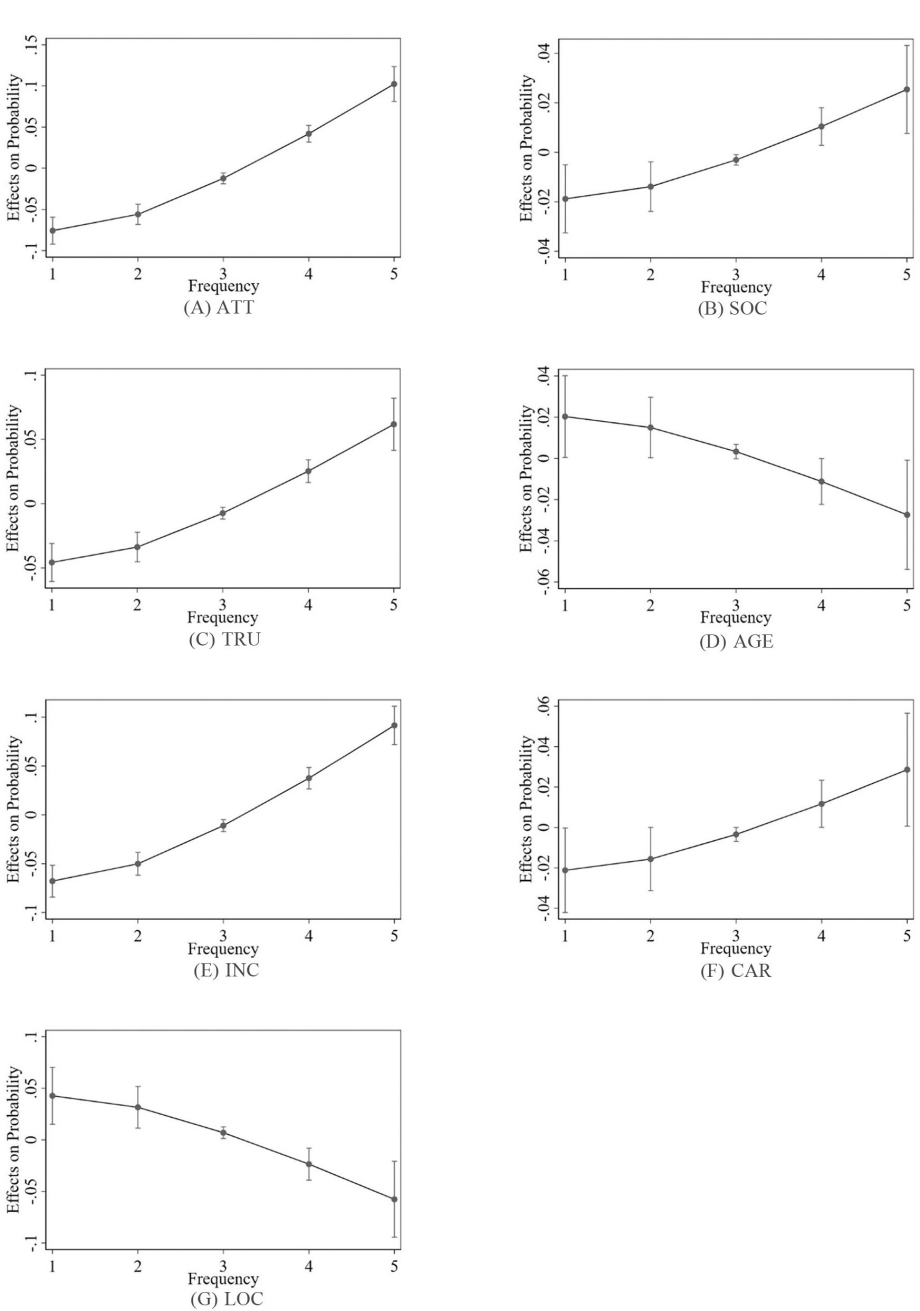
Average marginal effects at 95% confidence intervals before the epidemic.

**Figure 4:**
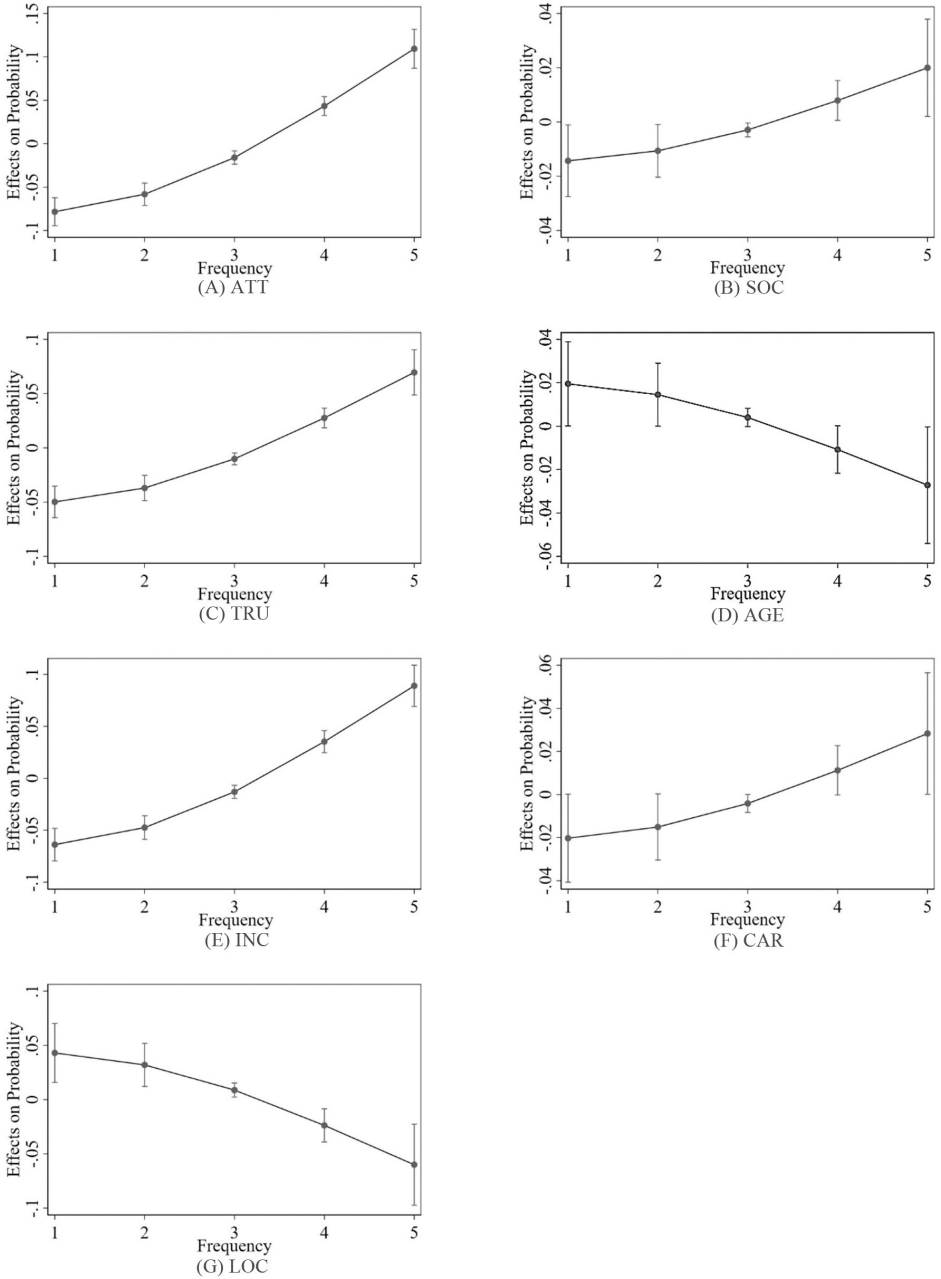
Average marginal effects at 95% confidence intervals after the epidemic.

The marginal effect of a previously fitted model was calculated by fixing the values of certain covariates and integrating over the remaining covariates. This effect demonstrated how the dependent variable would alter when a particular independent variable changed, with all other covariates held constant (35). To calculate the Average Marginal Effect (AME), the marginal effect of each variable was calculated for each observation, whilst taking into consideration any covariates, and then the average was determined. Based on the introduction to the regression model method given in results of ordered probit and ordered logit models section, the coefficient estimation of a variable in the regression-result table reflects the impact of the variable on the explained variables. However, the regression models considered in this study are non-linear. The line graphs of the marginal effects of all the factors are presented in Figures 3, 4. The predicted marginal values of seven independent variables before and after the epidemic are presented in Tables 4, 5. These values can be used to explore the influence of changes in independent variables on the changes in dependent variables and analyze and compare the size of the predicted marginal value of the dependent variable in different situations.

**Table 4:** Marginal effects of different factors before the epidemic.

		**Margin**	**Std.Err**	**z**	***P*>|z|**	**[95% Conf. Interval]**	
ATT	1	−0.0755^***^	0.0083	−9.11	0.000	−0.0917	−0.0592
	2	−0.0559^***^	0.0062	−8.96	0.000	−0.0682	−0.0437
	3	−0.0128^***^	0.0034	−3.75	0.000	−0.0195	−0.0061
	4	0.0417^***^	0.0052	8.06	0.000	0.0316	0.0518
	5	0.1025^***^	0.0109	9.42	0.000	0.0812	0.1238
SOC	1	−0.0204^***^	0.0070	−2.90	0.004	−0.0341	−0.0066
	2	−0.0151^***^	0.0051	−2.95	0.003	−0.0251	−0.0051
	3	−0.0035^***^	0.0011	−3.04	0.002	−0.0057	−0.0012
	4	0.0113^***^	0.0039	2.90	0.004	0.0037	0.0189
	5	0.0277^***^	0.0091	3.04	0.002	0.0098	0.0455
TRU	1	−0.0476^***^	0.0076	−6.28	0.000	−0.0625	−0.0328
	2	−0.0353^***^	0.0059	−5.95	0.000	−0.0469	−0.0237
	3	−0.0081^***^	0.0024	−3.32	0.001	−0.0128	−0.0033
	4	0.0263^***^	0.0046	5.78	0.000	0.0174	0.0352
	5	0.0647^***^	0.0105	6.17	0.000	0.0441	0.0852
AGE	1	0.0196^*^	0.0101	1.95	0.052	−0.0001	0.0394
	2	0.0145^*^	0.0075	1.94	0.053	−0.0002	0.0293
	3	0.0033^*^	0.0018	1.83	0.068	−0.0002	0.0069
	4	−0.0108^*^	0.0057	−1.92	0.055	−0.0219	0.0003
	5	−0.0267^**^	0.0136	−1.96	0.050	−0.0533	−0.0001
INC	1	−0.0662^***^	0.0082	−8.03	0.000	−0.0824	−0.0501
	2	−0.0491^***^	0.0060	−8.17	0.000	−0.0609	−0.0373
	3	−0.0112^***^	0.0031	−3.67	0.000	−0.0172	−0.0052
	4	0.0366^***^	0.0056	6.59	0.000	0.0257	0.0475
	5	0.0899^***^	0.0101	8.94	0.000	0.0702	0.1096
CAR	1	−0.0223^**^	0.0106	−2.10	0.036	−0.0431	−0.0014
	2	−0.0165^**^	0.0080	−2.07	0.039	−0.0321	−0.0009
	3	−0.0038^**^	0.0018	−2.06	0.039	−0.0074	−0.0002
	4	0.0123^**^	0.0059	2.07	0.038	0.0007	0.0239
	5	0.0302^**^	0.0143	2.12	0.034	0.0022	0.0582
LOC	1	0.0405^***^	0.0140	2.88	0.004	0.0129	0.0680
	2	0.0300^***^	0.0103	2.91	0.004	0.0098	0.0502
	3	0.0069^**^	0.0029	2.38	0.017	0.0012	0.0125
	4	−0.0224^***^	0.0079	−2.84	0.004	−0.0378	−0.0069
	5	−0.0550^***^	0.0189	−2.91	0.004	−0.0919	−0.0180

^*^p < 0.1; ^**^p < 0.05; ^***^p < 0.01.

**Table 5:** Marginal effects of different factors after the epidemic.

		**Margin**	**Std.Err**	**z**	***P*>|z|**	**[95% Conf. Interval]**	
ATT	1	−0.0778^***^	0.0082	−9.53	0.000	−0.0938	−0.0618
	2	−0.0582^***^	0.0065	−8.91	0.000	−0.0710	−0.0454
	3	−0.0166^***^	0.0040	−4.16	0.000	−0.0244	−0.0088
	4	0.0433^***^	0.0056	7.77	0.000	0.0324	0.0542
	5	0.1093^***^	0.0116	9.46	0.000	0.0867	0.1320
SOC	1	−0.0145^**^	0.0067	−2.15	0.031	−0.0277	−0.0013
	2	−0.0108^**^	0.0050	−2.18	0.029	−0.0206	−0.0011
	3	−0.0031^**^	0.0013	−2.29	0.022	−0.0057	−0.0004
	4	0.0081^**^	0.0038	2.15	0.032	0.0007	0.0154
	5	0.0204^**^	0.0092	2.22	0.026	0.0024	0.0383
TRU	1	−0.0507^***^	0.0074	−6.86	0.000	−0.0651	−0.0362
	2	−0.0379^***^	0.0060	−6.30	0.000	−0.0497	−0.0261
	3	−0.0108^***^	0.0029	−3.75	0.000	−0.0165	−0.0052
	4	0.0282^***^	0.0047	5.99	0.000	0.0190	0.0374
	5	0.0712^***^	0.0107	6.63	0.000	0.0501	0.0922
AGE	1	0.0192^*^	0.0099	1.94	0.052	−0.0002	0.0385
	2	0.0143^*^	0.0074	1.93	0.053	−0.0002	0.0289
	3	0.0041^*^	0.0022	1.85	0.064	−0.0002	0.0084
	4	−0.0107^*^	0.0056	−1.91	0.057	−0.0216	0.0003
	5	−0.0269^**^	0.0138	−1.96	0.050	−0.0539	0.0000
INC	1	−0.0626^***^	0.0080	−7.85	0.000	−0.0782	−0.0470
	2	−0.0468^***^	0.0058	−8.06	0.000	−0.0582	−0.0354
	3	−0.0134^***^	0.0032	−4.17	0.000	−0.0196	−0.0071
	4	0.0348^***^	0.0054	6.42	0.000	0.0242	0.0455
	5	0.0879^***^	0.0102	8.66	0.000	0.0680	0.1078
CAR	1	−0.0208^**^	0.0104	−2.00	0.045	−0.0411	−0.0005
	2	−0.0155^**^	0.0078	−1.98	0.047	−0.0309	−0.0002
	3	−0.0044^**^	0.0022	−1.99	0.046	−0.0088	−0.0001
	4	0.0116^**^	0.0058	1.98	0.048	0.0001	0.0230
	5	0.0292^**^	0.0144	2.02	0.043	0.0009	0.0575
LOC	1	0.0418^***^	0.0138	3.02	0.002	0.0147	0.0689
	2	0.0313^***^	0.0102	3.08	0.002	0.0114	0.0512
	3	0.0089^***^	0.0034	2.63	0.008	0.0023	0.0156
	4	−0.0233^***^	0.0078	−2.98	0.003	−0.0385	−0.0080
	5	−0.0587^***^	0.0191	−3.07	0.002	−0.0962	−0.0213

Std.Err is in the brackets.

^*^p < 0.1; ^**^p < 0.05; ^***^p < 0.01.

The positive marginal effect indicates that as the value of a factor increases, the possibility of consumers choosing to use the ride-hailing will also increase, and the greater the value is, the greater the possibility will be. As shown in Table 5 before the epidemic, the factor TRU “passengers' trust and loyalty” had a negative marginal effect on the frequency of use, i.e., less than once a month, 1–3 times a month, and 1–2 times a week, and had a positive marginal effect on the usage frequency, i.e., 3–4 times a week and more than five times a week, and the marginal effect for more than five times a week was the largest at 0.0647, indicating that when the other factors remained unchanged, for a one-unit increase in the passengers' trust, the possibility of consumers choosing to use the ride-hailing five or more times a week increased by 6.47%, and the possibility of choosing less than once a month decreased by 4.76%. After the epidemic, when other factors remained unchanged, for a one-unit increase in the passengers' trust, the probability that consumers would choose to use the ride-hailing five or more times a week increased by 7.12%, while the probability to use it less than once a month decreased by 5.07%. Thus, the more passengers trusted the service, the more frequently it was used, and vice versa.

Similarly, before and after the epidemic, the four factors, including the ATT, SOC, INC, and CAR, had a negative marginal effect on the frequency of use, i.e., less than once a month, 1–3 per month, and 1–2 times a week, and had a positive marginal effect on the usage frequency, i.e., 3–4 times a week and more than five times a week, and the factor “attitudes to environmental protection and commuting” after the epidemic had the largest marginal effect at 0.1093, indicating that when the other factors remained unchanged, for a one-unit increase in the attitudes to environmental protection and commuting, the possibility of consumers choosing to use the ride-hailing five times or more per week increased by 10.93%. The results also show that the more private cars people owned, the higher the usage frequency of ride-hailing would be. This may be due to the fact that people who own private cars are more inclined to travel by cars, and thus they have a higher acceptance of ride-hailing services. Also, when it is not possible to use a private car to travel, they are more willing to choose ride-hailing services rather than other traveling methods, such as public transportation.

Contrary to the above factors, both before and after the epidemic, the marginal effect of the age and residential location in the urban area on the frequency of use, i.e., less than once a month, 1–3 times a month, and 1–2 times a week, was positive, the marginal effect on the usage frequency, i.e., 3–4 times a week and more than five times a week, was negative. When the other factors remained unchanged, for a one-unit increase in the residential location in the urban area before the epidemic, the possibility of consumers choosing to use the ride-hailing five or more times a week decreased by 5.50%, and the possibility of choosing it less than once a month increased by 4.05%.

After the epidemic, when the other factors remained unchanged, for a one-unit increase in the location and convenience of car usage before the epidemic, the probability that consumers would choose to use the ride-hailing five or more times per week decreased by 5.87%, and the probability of choosing it once a month increased by 4.18%. This result shows that people who live outside the urban area and are farther away from the urban area use ride-hailing services less frequently.

The possible reason for this result can be that the farther away from the urban area people are, the less the supply of ride-hailing services may be. Also, compared to the urban areas, the convenience of obtaining the ride-hailing services in rural and suburban areas can be slightly worse, which can make people in that areas be more inclined not to use the ride-hailing or to use them less often. Furthermore, the older the person is, the lower the usage frequency of ride-hailing will be. Based on the descriptive statistics, users who had used ride-hailing services were mainly between the ages of 18 and 34, and the younger generation was the majority. This age group also accounted for the highest proportion of participants who had used the ride-hailing services. The authors believe that there are two possible reasons for this result: (1) people in this age group have higher travel needs, and (2) they are highly adaptable to technological changes and are more likely to accept new technologies.

## 4. Discussion

In this study, the data obtained from the online questionnaires are used to investigate the factors affecting the usage frequency of ride-hailing services in China. Many studies have found the impact of the COVID-19 pandemic on passengers' ride-hailing frequency. It has been established by Morshed et al. (31) that the COVID-19 pandemic has had a significant impact on the ride-hailing market, leading to a decrease in its popularity as a transportation option. The recent COVID-19 pandemic has caused a significant decrease in the revenue of on-demand ride-hailing services due to the fear of infection in shared vehicles (36, 37). A study conducted in Chicago revealed a substantial decline in the number of ride-hailing trips when compared to those using private cars during the COVID-19 pandemic (36). Nguyen-Phuoc et al. (38) concluded that self-efficacy has the most significant influence on self-protective behaviors among ride-hailing passengers during the COVID-19 pandemic. According to the regression results of this study, it can be concluded that the travelers' usage frequency of ride-hailing services is highly correlated with the following seven factors: ATT, SOC, TRU, AGE, INC, PRIVATE, and LOC. Besides, based on the result comparison of different models, the individual's perception of the EPI does not have a significant impact on the usage frequency of ride-hailing services.

Specifically, the factor passenger trust and loyalty are positively related to the frequency of use of ride-hailing services. The marginal effect diagram in marginal effect analysis section shows that the more passengers trust the service, the higher the frequency of use of ride-hailing services will be, and vice versa. Similarly, consistent with our findings, Nguyen-Phuoc et al. (39) demonstrate that perceived benefits, perceived sales promotion and perceived service quality are all direct contributors to passenger satisfaction and loyalty, with perceived service quality being the most influential factor (39). Nguyen-Phuoc et al. (11) examined the direct and indirect effects of elements impacting the loyalty of ride-hailing and conventional taxi users. Ma et al. (40) found that trust in drivers has a positive influence on users' trust and attitude toward the platform. Trust and implicit cost have been found to have a positive influence on e-loyalty relate to ride-hailing (41). Chinese passengers view ride-hailing services as less secure than traditional taxis, and women are more likely to be affected by the perceived lack of security than men (42).

It is imperative to increase the convenience, security, and popularity of ride-hailing services in order to build trust and loyalty among passengers and thus, increase the number of people using the service. To this end, it is necessary to promote complaints and feedback mechanisms for improvement; improve online payment methods; implement practical measures to ensure the safety of passengers; and provide transparent information to enhance corporate image and value, so that passengers can continue to choose ride-hailing services and recommend them to others.

Studies have shown a positive correlation between social life and technology usage and the frequency of use of ride-hailing services, which is in line with the findings of prior studies. Additionally, attitudes toward environmental protection and commuting are positively associated with the frequency of use of ride-hailing services. Urban residential location, however, is negatively correlated with the frequency of use of ride-hailing services, which may be due to the lower availability of ride-hailing services in these areas. Age and frequency of use of ride-hailing services are also negatively correlated, indicating that young people are the main demographic using ride-hailing. Finally, the number of private cars is positively correlated with the frequency of use of ride-hailing services, as those who own private cars are more likely to use ride-hailing services when they cannot use their own vehicles.

**Figure 5:**
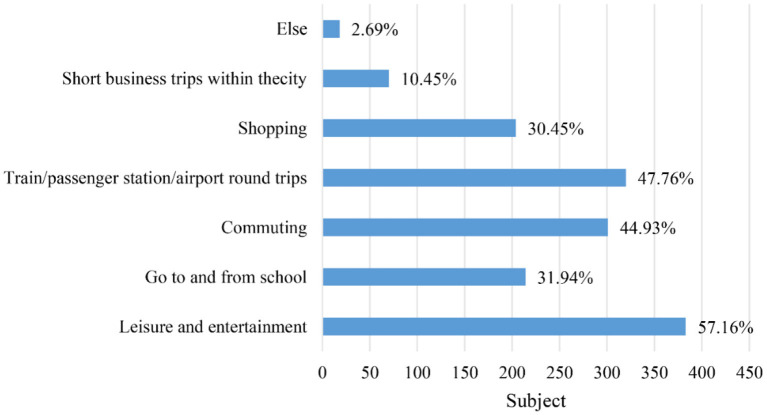
Purpose of travel before the epidemic.

**Figure 6:**
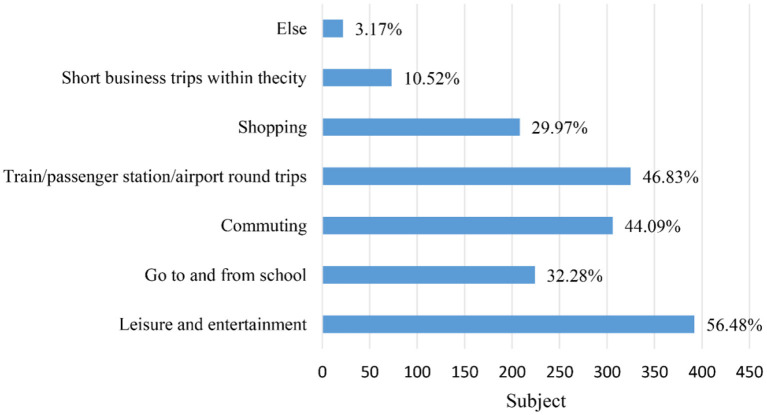
Purpose of travel after the epidemic.

**Figure 7:**
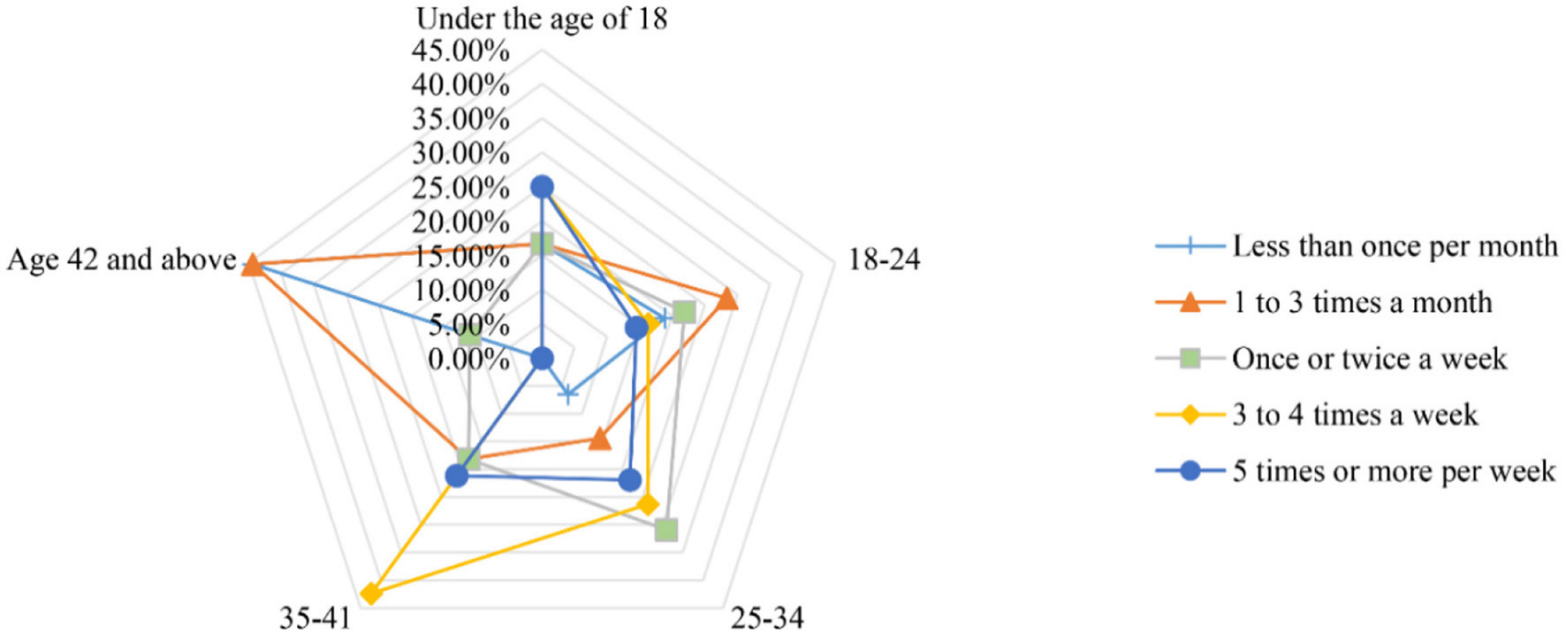
Radar charts of the age group and frequency of use of ride-hailing services before the epidemic.

**Figure 8:**
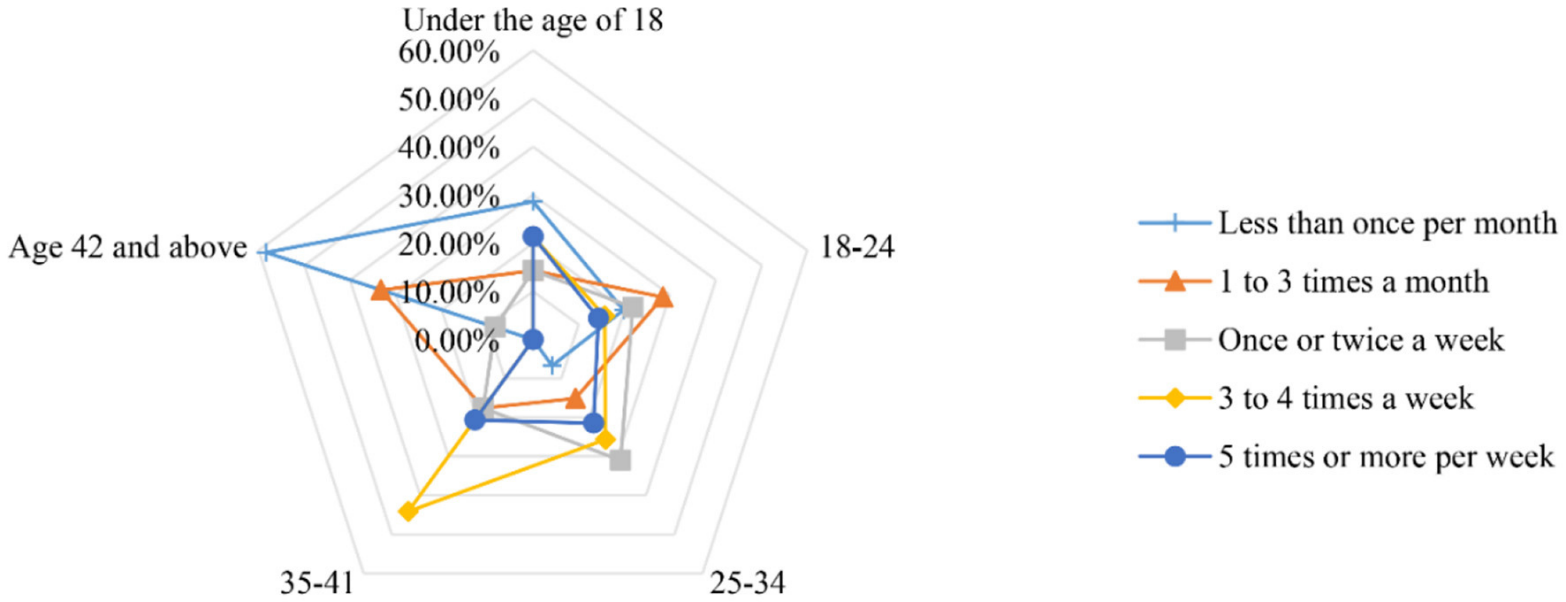
Radar charts of the age group and frequency of use of ride-hailing services after the epidemic.

The survey results revealed that the majority of ride-hailing trips (about 60%) were for leisure and entertainment. This was followed by trips to/from train/passenger station/airport (about 50%) and commuting (about 45%). The figures in Figures 5, 6 illustrate the trip purpose in the two questionnaires. Ride-hailing services offer a convenient solution to the problems associated with personal driving (e.g., parking and drinking) and public transportation (e.g., time control and comfort). This could explain why ride-hailing is so popular for leisure and entertainment. Figures 7, 8 show the usage frequency and age range of ride-hailing users, respectively. It can be seen that the majority of users choose ride-hailing 1–3 times a month and 1–2 times a week. The highest usage frequency of ride-hailing was among the 18–34 age group, which is in line with the previous conclusion.

## 5. Conclusion

This study conducted a two-stage survey among the same group of passengers to investigate the factors influencing people's usage of ride-hailing services in the pre-epidemic and post-epidemic phases. This research was distinct from existing literature in that it included discussions on two additional factors: the epidemics and the passenger trust, and their impacts on the frequency of use of ride-hailing services were quantitatively studied. The survey acquired information on participants, including their geographic area, lifestyle, technology use, personal attitudes, and social economy, which was then subjected to an Exploratory Factor Analysis to develop a usage frequency model of ride-hailing. Model estimation results revealed that the passenger trust and loyalty, social life and technology use, attitudes to environmental protection and commuting, age, personal income, number of private cars, and residential location in the urban area had a significant impact on the usage frequency of ride-hailing services. The results indicated that in the long run, the epidemic would have a slight impact on the usage frequency of ride-hailing.

This study provides beneficial information for firms and executives when analyzing ride-hailing models. To guarantee passenger security during the pandemic, enterprises and platforms should execute practical safeguards, such as mandating drivers to don face masks, measure and transmit body temperature data, frequently disinfect vehicles, and open windows for ventilation. The reliable statistical models used in this research are also beneficial for future research and provide useful advice for regulators, managers, and enterprises.

This initial study was limited by sample size, encompassing only 670 valid cases. Future research should aim to expand the scope of the study by increasing the sample size and collecting data from a more diverse geographical area, employing various techniques such as machine learning. Future research should extend this study by assessing different causal relationship structures, and contrasting the magnitude of each endogenous variable's effect on ride-hailing and other behaviors. Moreover, to address the limitations of the current analyses, preference heterogeneity should be incorporated in the model estimation, thereby allowing us to gain insight into the individual decision-making process.

## Data availability statement

The original contributions presented in the study are included in the article/Supplementary material, further inquiries can be directed to the corresponding authors.

## Author contributions

SL: writing—original draft and supervision. YJ: software and writing—original draft. XY: data curation, validation, and methodology. HD: conceptualization. TZ: language polishing and data analysis. All authors contributed to the article and approved the submitted version.

## References

[1] Flor M Ortuño A. Ride-hailing services: competition or complement to public transport to reduce accident rates. The case of Madrid. Front Psychol. (2022) 13:951258. 10.3389/fpsyg.2022.951258 35967705 PMC9363903

[2] Guidon S Wicki M Bernauer T. Transportation service bundling–For whose benefit? Consumer valuation of pure bundling in the passenger transportation market. Transp Res Part A Policy Practice. (2020) 131:91–106. 10.1016/j.tra.2019.09.023

[3] Brown AE. Who and where rideshares? Rideshare travel and use in Los Angeles. Transp Res Part A Policy Practice. (2020) 136:120–34. 10.1016/j.tra.2020.04.001

[4] Morris EA Blumenberg E. Does lacking a car put the brakes on activity participation? Private vehicle access and access to opportunities among low-income adults. Transp Res Part A Policy Practice. (2020) 136:375–97. 10.1016/j.tra.2020.03.021

[5] Shaheen S Chan N Bansal A. Shared Mobility: A Sustainability and Technologies Workshop: Definitions, Industry Developments, and Early Understanding (2015).

[6] She S Xu H Wu Z Tian Y. (2020). Dimension, content, and role of platform psychological contract: based on online ride-hailing users. Front Psychol. (2097) 11:2097. 10.3389/fpsyg.2020.02097 33101102 PMC7554242

[7] Schaller B. Can sharing a ride make for less traffic? Evidence from Uber and Lyft and implications for cities. Transp Policy. (2021) 102:1–10. 10.1016/j.tranpol.2020.12.015

[8] Pew Research Center. Methodology: The American Trends Panel Survey Methodology. https://www.pewforum.org/2019/06/11/methodology-25/ (2020). p. 1–4.

[9] Septiani R Handayani PW. Factors that affecting behavioral intention in online transportation service: case study of GO-JEK. Procedia Comput Sci. (2017) 124:504–12. 10.1016/j.procs.2017.12.18334855753

[10] Huynh TLD Vo AKH Nguyen THH Le Nguyen VB Ho NNH. What makes us use the shared mobility model? Evidence from Vietnam. Econ Anal Policy. (2020) 66:1–13. 10.1016/j.eap.2020.02.007

[11] Nguyen-Phuoc DQ Oviedo-Trespalacios O Vo NS Le PT. How does perceived risk affect passenger satisfaction and loyalty towards ride-sourcing services?. Transp Res Part D Transp Environ. (2021) 97, 102921. 10.1016/j.trd.2021.102921

[12] Mitra SK Bae Y. Use of ride-hailing services among older adults in the United States. Transp Res Record. (2019) 2673:700–10. 10.1177/036119811983551132199581

[13] Alemi F Circella G Mokhtarian P Handy S. Exploring the latent constructs behind the use of ridehailing in California. J Choice Model. (2018) 29:47–62. 10.1016/j.jocm.2018.08.003

[14] Murphy C Feigon S. Shared Mobility and the Transformation of Public Transit. (2016). 10.17226/23578

[15] Alemi F Circella G Mokhtarian P Handy S. What drives the use of ridehailing in California? Ordered probit models of the usage frequency of Uber and Lyft. Trans Res Part C: Emerg Technol. (2019) 102:233–48. 10.1016/j.trc.2018.12.016

[16] Zheng Z Zhang J Zhang L Li M Rong P. Understanding the impact of the built environment on ride-hailing from a spatio-temporal perspective: a fine-scale empirical study from China. Cities. (2022) 126: 103706. 10.1016/j.cities.2022.103706

[17] Gomez J Aguilera-Garcia A Dias FF Bhat CR. Adoption and frequency of use of ride-hailing services in a European city: the case of Madrid. Transp Res Part C Emerg Technol. (2021) 131:103359. 10.1016/j.trc.2021.103359

[18] Guest L. A study of brand loyalty. J Appl Psychol. (1944) 28:16–27. 10.1037/h0053554

[19] Boulding W Kalra A Staelin R Zeithaml VA. A dynamic process model of service quality: from expectations to behavioral intentions. J Market Res. (1993) 30:2–27. 10.1177/002224379303000102

[20] Barajas JM Brown A. Not minding the gap: Does ride-hailing serve transit deserts?. J Trans Geograph. (2021) 90:102918. 10.1016/j.jtrangeo.2020.102918

[21] Tirachini A. Ride-hailing, travel behaviour and sustainable mobility: an international review. Transportation. (2020) 47:2011–47. 10.1007/s11116-019-10070-2

[22] Lee CKH. Antecedents of consumer loyalty in ride-hailing. Transp Res Part F Traffic Psychol Behav. (2021) 80:14–33. 10.1016/j.trf.2021.03.016

[23] Groß M. Impediments to mobile shopping continued usage intention: A trustrisk-relationship. J Retail Consum Serv. (2016) 33:109–19. 10.1016/j.jretconser.2016.08.013

[24] Marriott HR. Exploring consumers perceived risk and trust for mobile shopping: a theoretical framework and empirical study. J Retail Consumer Serv. (2018) 42, 133–146. 10.1016/j.jretconser.2018.01.017

[25] Elnadi M Gheith MH. What makes consumers reuse ride-hailing services? An investigation of Egyptian consumers' attitudes towards ride-hailing apps. Travel Behav Soc. (2022) 29:78–94. 10.1016/j.tbs.2022.06.002

[26] Aw ECX Basha NK Ng SI Sambasivan M. To grab or not to grab? The role of trust and perceived value in on-demand ridesharing services. Asia Pacific J Mark Logist. (2019) 31:1442–65. 10.1108/APJML-09-2018-0368

[27] Fauzi AA Sheng ML. Ride-hailing apps' continuance intention among different consumer groups in Indonesia: the role of personal innovativeness and perceived utilitarian and hedonic value. Asia Pacific J Mark Logist. (2021) 33:1195–219. 10.1108/APJML-05-2019-0332

[28] Weng GS Zailani S Iranmanesh M Hyun SS. Mobile taxi booking application service's continuance usage intention by users. Transp Res Part D Transp Environ. (2017) 57:207–16. 10.1016/j.trd.2017.07.023

[29] Zhang J. Transport policymaking that accounts for COVID-19 and future public health threats: a PASS approach. Transp Policy. (2020) 99:405–18. 10.1016/j.tranpol.2020.09.009 32952316 PMC7486869

[30] Monahan T Lamb CG. Transit's downward spiral: assessing the social-justice implications of ride-hailing platforms and COVID-19 for public transportation in the US. Cities. (2022) 120:103438. 10.1016/j.cities.2021.103438

[31] Morshed SA Khan SS Tanvir RB Nur S. Impact of COVID-19 pandemic on ride-hailing services based on large-scale Twitter data analysis. J Urban Manage. (2021) 10:155–65. 10.1016/j.jum.2021.03.002

[32] Kotler P. Behavioral models for analyzing buyers. J Mark. (1965) 29:37–45. 10.1177/002224296502900408

[33] Maccallum RC Widaman KF Zhang S Hong S. Sample size in factor analysis. Psychol Methods. (1999) 4:84–99. 10.1037/1082-989X.4.1.84

[34] Velicer WF Fava JL. Affects of variable and subject sampling on factor pattern recovery. Psychol Methods. (1998) 3:231–51. 10.1037/1082-989X.3.2.231

[35] Baum CF. An Introduction to Modern Econometrics Using Stata. Stata Press (2006).

[36] Choi TM. On-demand ride-hailing service platforms with hired drivers during coronavirus (COVID-19) outbreak: can blockchain help? IEEE Trans Eng Manage. (2022) 10.1109/TEM.2021.3131044

[37] Silveira-Santos T González ABR Rangel T Pozo RF Vassallo JM. Were ride-hailing fares affected by the COVID-19 pandemic? Empirical analyses in Atlanta and Boston. Transportation. (2022) 1–32. 10.1007/s11116-022-10349-x 36407885 PMC9649021

[38] Nguyen-Phuoc DQ Su DN Dinh MTT Newton JDA. Passengers' self-protective intentions while using ride-hailing services during the COVID-19 pandemic. Safety Sci. (2023) 157:105920. 10.1016/j.ssci.2022.105920 36091924 PMC9444896

[39] Nguyen-Phuoc DQ Su DN Tran PTK Le DTT. Factors influencing customer's loyalty towards ride-hailing taxi services—a case study of Vietnam. Transp Res Part A Policy Practice. (2020) 134:96–112. 10.1016/j.tra.2020.02.008

[40] Ma L Zhang X Ding X Wang G. Risk perception and intention to discontinue use of ride-hailing services in China: taking the example of DiDi Chuxing. Transp Res Part F Traffic Psychol Behav. (2019) 66:459–70. 10.1016/j.trf.2019.09.021

[41] Hou T Cheng X. The role of transaction cost and trust in e-loyalty: a mixed-methods study of ride-sharing. Inf Technol People. (2020) 34:1018–38. 10.1108/ITP-01-2020-0005

[42] Liu Y Gao Q. Chinese passengers' security perceptions of ride-hailing services: an integrated approach combining general and situational perspectives. Travel Behav Society. (2022) 26:250–69. 10.1016/j.tbs.2021.10.009

