# Supplementary material for: Understanding the motivational mechanisms behind the usage frequency of ride-hailing during COVID-19 pandemic
Source: Front Public Health. 2023 Jan 26;10:1097885. doi: 10.3389/fpubh.2022.1097885 (PMC9909234; doi:10.3389/fpubh.2022.1097885)
Supplement: Supplementary file 1 [file Data_Sheet_1.pdf]

Appendix

Table A1. Introduction to questionnaire variables before the epidemic (based on the average trip)

Factor	Offered descriptions in the questionnaire
TRU_1	I would like to describe the pleasant experience of using the ride-hailing service to my relatives and friends.
TRU_2	I plan to use ride-hailing services frequently in the future.
TRU_3	I think the evaluation information provided by the ride-hailing platform is true and effective.
TRU_4	I would like to recommend ride-hailing services to other people.
TRU_5	I think ride-hailing services can guarantee my health and safety.
TRU_6	I think the complaint feedback mechanism of ride-hailing services is trustworthy.
TRU_7	I think the payment method for ride-hailing services is very safe.
SOC_1	I think that with the usage of smartphones, travel has become more convenient than ever.
SOC_2	No matter where I go, it is essential for me that my mobile device can connect to WIFI and/or 3G/4G.
SOC_3	Usage frequency of social media.
SOC_4	I like to try various modes of travel, such as walking, riding, and cycling.
SOC_5	I have a strong interest in traveling to other provinces, cities, or countries.
SOC_6	I like to use my smartphone and mobile apps to plan my trip.
SOC_7	Social media (such as Weibo) makes my life more interesting.
SOC_8	I am very popular and get along very well with my friends.
SOC_9	Technology has brought many solutions but also brought certain problems.
SOC_10	I like to try new and different things.
ATT_1	For me, because I am just an individual, trying to be more environmentally friendly is meaningless to me.
ATT_2	All kinds of climate changes are part of the natural cycle and have little to do with human activities.
ATT_3	My schedule makes it difficult or impossible for me to use public transportation.
ATT_4	Most of the time, I have no choice but to drive.
ATT_5	Learning how to use new technologies is often frustrating.
ATT_6	I spend a lot of money on commuting.
ATT_7	The fuel prices should be increased to reduce the negative impact on the environment.
ATT_8	Fuel prices affect my choice of daily travel mode.
ATT_9	My commute is usually pleasant.
ATT_10	It is hard to use my commuting time to do something that I think is meaningful.

Table A2. Introduction to questionnaire variables after the epidemic (based on the average trip)

Factor	Offered descriptions in the questionnaire
TRU'_1	After the epidemic, I am still willing to recommend ride-hailing services to other people.
TRU'_2	After the epidemic, I still feel that ride-hailing can ensure my safety and health.
TRU'_3	After the epidemic, I plan to use ride-hailing services frequently.
TRU'_4	After the epidemic, I will continue to use ride-hailing services.
TRU'_5	After the epidemic, I think the evaluation information provided by the ride-hailing platform is true and effective.
TRU'_6	After the epidemic, I think the complaint feedback mechanism of ride-hailing services is trustworthy.
SOC_1	I think that with the usage of smartphones, travel has become more convenient than ever.
SOC_2	No matter where I go, it is essential for me that my mobile device can connect to WIFI and/or 3G/4G.
SOC_3	Usage frequency of social media.
SOC_4	I like to try various modes of travel, such as walking, riding, and cycling.
SOC_5	I have a strong interest in traveling to other provinces, cities, or countries.
SOC_6	I like to use my smartphone and mobile apps to plan my trip.
SOC_7	Social media (such as Weibo) makes my life more interesting.
SOC_8	I am very popular and get along very well with my friends.
SOC_9	Technology has brought many solutions but also brought certain problems.
SOC_10	I like to try new and different things.
ATT_1	For me, because I am just an individual, trying to be more environmentally friendly is meaningless to me.
ATT_2	All kinds of climate changes are part of the natural cycle and have little to do with human activities.
ATT_3	My schedule makes it difficult or impossible for me to use public transportation.
ATT_4	Most of the time, I have no choice but to drive.
ATT_5	Learning how to use new technologies is often frustrating.
ATT_6	I spend a lot of money on commuting.
ATT_7	The fuel prices should be increased to reduce the negative impact on the environment.
ATT_8	Fuel prices affect my choice of daily travel mode.
ATT_9	My commute is usually pleasant.
ATT_10	It is hard to use my commuting time to do something that I think is meaningful.
EPI_1	The epidemic has made me feel that going out can be very dangerous.
EPI_2	The epidemic has made me feel it can be more difficult to travel.
EPI_3	The outbreak of COVID-19 has significantly reduced my usage frequency of ride-hailing.

Table A3. Rotated component matrix before the epidemic

Variable	Component		
	SOC	ATT	TRU
SOC_1	**0.747**	-0.140	0.102
SOC_2	**0.715**	-0.081	0.130
SOC_3	**0.714**	-0.054	0.079
SOC_4	**0.614**	0.162	0.185
SOC_5	**0.609**	0.183	0.183
SOC_6	**0.596**	0.146	0.264
SOC_7	**0.592**	0.140	0.253
SOC_8	**0.541**	0.270	0.355
SOC_9	**0.505**	0.395	0.152
SOC_10	**0.500**	0.203	0.323
ATT_1	0.038	**0.823**	0.239
ATT_2	0.055	**0.816**	0.223
ATT_3	0.080	**0.699**	0.266
ATT_4	0.058	**0.685**	0.343
ATT_5	0.080	**0.676**	0.215
ATT_6	0.159	**0.661**	0.411
TRU_1	0.255	0.304	**0.751**
TRU_2	0.183	0.355	**0.676**
TRU_3	0.263	0.319	**0.663**
TRU_4	0.259	0.319	**0.657**
TRU_5	0.283	0.300	**0.645**
TRU_6	0.265	0.352	**0.606**
TRU_7	0.447	0.199	**0.566**

Table A4. Rotated component matrix after the epidemic

Variable	Component			
	ATT	SOC	TRU	EPI
ATT_1	**0.816**	-0.001	0.176	0.125
ATT_2	**0.800**	0.013	0.153	0.165
ATT_3	**0.716**	0.156	0.247	0.158
ATT_4	**0.700**	0.052	0.157	0.187
ATT_5	**0.698**	0.041	0.207	0.220
ATT_6	**0.690**	0.048	0.099	0.176
ATT_7	**0.574**	0.235	0.176	0.121
ATT_8	**0.558**	0.275	0.311	0.109
ATT_9	**0.549**	0.231	0.235	0.157
ATT_10	**0.545**	0.416	0.246	-0.024
SOC_1	-0.125	**0.737**	0.100	0.094
SOC_2	-0.086	**0.684**	0.121	0.173
SOC_3	-0.062	**0.673**	0.098	0.107
SOC_4	0.220	**0.633**	0.166	0.029
SOC_5	0.192	**0.617**	0.161	0.094
SOC_6	0.216	**0.604**	0.148	0.096
SOC_7	0.180	**0.593**	0.239	0.054
SOC_8	0.345	**0.564**	0.294	0.040
SOC_9	0.274	**0.523**	0.239	0.066
SOC_10	0.388	**0.505**	0.271	0.026
TRU_1	0.269	0.251	**0.719**	0.134
TRU_2	0.292	0.300	**0.713**	0.067
TRU_3	0.346	0.196	**0.666**	0.089
TRU_4	0.084	0.289	**0.607**	0.172
TRU_5	0.438	0.286	**0.527**	0.137
TRU_6	0.456	0.281	**0.502**	0.098
EPI_1	0.327	0.182	0.174	**0.718**
EPI_2	0.382	0.163	0.116	**0.602**
EPI_3	0.372	0.203	0.168	**0.543**
